# Response to “Stronger Evaluations are Needed for Interventions to Improve Health Worker Performance”

**DOI:** 10.4269/ajtmh.24-0539a

**Published:** 2024-10-29

**Authors:** Ruth Ashton, Joshua Yukich, Lawrence Barat

**Affiliations:** School of Public Health and Tropical Medicine, Tulane University, New Orleans, Louisiana Tropical Health, New Orleans, Louisiana E-mail: rashton@tulane.edu; School of Public Health and Tropical Medicine, Tulane University, New Orleans, Louisiana Tropical Health, New Orleans, Louisiana E-mail: jyukich@tulane.edu; Independent Consultant, Provincetown, Massachusetts E-mail: larrybarat@yahoo.com

Dear Editor,

We thank Dr. Rowe for his comments, and agree that observational, retrospective studies such as our evaluation of Outreach, Training, and Supportive Supervision (OTSS) have limitations.^1^^,^^2^ These limitations include those we noted extensively in the discussion section of our article, and those which Dr. Rowe highlights. We fully agree that observational studies cannot provide the kind of robust evidence base that a series of large, well-designed cluster randomized controlled trials could, or even that which could be established by better controlled quasi-experimental studies.

Certainly, as public health researchers we strongly favor future investments in such research. However, we would also caution that OTSS-type strategies for supportive supervision of health care workers are unlikely to cause harm, and extensive practical experience in health care and other domains suggest that supervision and ongoing education are important to improving and maintaining performance. Consequently, the need for such expensive and time-consuming evidence generation to undergird these kinds of strategies is debatable. Furthermore, variation in health systems within and between countries mean that standardization of such strategies as necessary for controlled trials limit their generalizability and might make evidence generated from these trials less useful. In the context of ongoing funding constraints, we must be careful that desire for highest quality evidence is balanced with using available funds to support quality malaria prevention and case management in communities today.

For clarification, in response to Dr. Rowe’s point #6, we note that baseline values for summary outcome indicators assessed in regression models can be interpreted from Figure 1, and to point #1, additional models which retained all facilities receiving at least one visit are presented in supplementary tables.^2^ Furthermore, per point #5, while data quality assessment procedures for OTSS visit data were not discussed in Ashton et al.^2^ we note that the Impact Malaria project had extensive mechanisms to monitor and assess data quality, and that these processes are detailed elsewhere in the supplement.^2^^,^^3^ These mechanisms include use of digitized checklists loaded on tablet computers for data collection and quarterly data reviews at district level.

Despite their limitations, evaluation methods such as those used in this assessment also could be strengthened by: 1) ensuring that key information is included in supervision checklists (such as information on corrective actions taken that Dr. Rowe mentions), 2) further improving the completeness and quality of data entry, including increased use of digital data collection, and 3) using more systematic approaches for selecting facilities to be included in such analyses. As countries continue to scale up these approaches, thereby increasing the number of facilities that have received multiple OTSS visits, future analyses of these data will help clarify the effectiveness of this quality improvement approach.

Lastly, we note that while OTSS-like strategies are not the only approach to improve and maintain health worker performance in malaria case management, considering the persistent high malaria burden in many settings and increasing complexity of care resulting from growing drug and diagnostic resistance in *Plasmodium*, methods like OTSS which can help ensure high quality malaria care have never been more necessary.
